# What Are Lay Theories of Social Class?

**DOI:** 10.1371/journal.pone.0070589

**Published:** 2013-07-16

**Authors:** Michael E. W. Varnum

**Affiliations:** Department of Psychology, Peking University, Beijing, China; The University of New South Wales, Australia

## Abstract

Numerous studies have documented the effects of social class on psychological and behavioral variables. However, lay beliefs about how social class affects these dimensions have not been systematically tested. Studies 1 and 2 assessed lay beliefs about the association between social class and 8 variables (including psychological and behavioral tendencies and cognitive ability). Study 3 assessed lay beliefs about the Big five personality traits and social class, and study 4 reframed the 8 variables from study 1 in opposite terms and yielded similar results. Study 5 contained the variables framed as in both studies 1 and 4, and replicated those results suggesting that framing effects were not responsible for the effects observed. Interestingly, for the most part lay beliefs about social class did not differ as a function of participants’ own social class. In general people held relatively accurate and consistent stereotypes about the relationship between social class and well-being, health, intelligence, and neuroticism. In contrast lay beliefs regarding social class and reasoning styles, as well as relational, social, and emotional tendencies were less consistent and coherent. This work suggests that on the whole people’s beliefs about social class are not particularly accurate, and further that in some domains there are contradictory stereotypes about the consequences of social class.

## Introduction

Let me tell you about the very rich. They are different from you and me. - F. Scott Fitzgerald

The idea that those who are higher in social class differ from those who are lower in social class is perhaps as old as human civilization. In the West this discussion goes back at least to Plato [1]. In recent years social scientists, and social psychologists in particular, have turned their attention to the consequences of social class for psychological and behavior tendencies. Social class has been linked to differences in phenomena as diverse as social mimicry [2], patterns of visual attention [3], causal explanation [3,4], and ethical behavior in real world settings [5]. Yet to date no studies have systematically explored how lay theories of social class fit with empirical findings. We may all know that the rich are different (to paraphrase Fitzgerald), but do we really know *how* they are different?

Although some previous research has suggested that social class stereotypes may be fairly accurate, at least those held by teachers when it comes to academic success and effort [6]. That is teachers beliefs regarding students from different social class backgrounds were significantly correlated with actual performance. At first glance these findings appear problematic as teachers are in a strong position to influence actual academic performance in a way that corresponds with their beliefs, similar to the classic demonstration of self-fulfilling prophecy, which also coincidentally took place in a classroom environment [7]. Further, stereotype threat has been shown to affect the academic performance of low-SES students [8]. However, the study was designed so that past academic success and student motivation were the criteria by which accuracy was judged, and the researchers found that teacher perceptions of individual students appeared to be more influenced by actual past performance than by stereotypes regarding social class. It should be noted though that teacher stereotypes about social class were not directly assessed (but rather were statistically inferred by testing parental income and education as predictors of teacher perceptions of individual students while controlling for actual past achievement and motivation) making interpretation of these results somewhat difficult. Further, although this work suggests that people may hold stereotypes linking higher social class with greater intellectual ability or achievement, it does not speak to whether such views are shared by the general population.

Other work has explored dimensions that underlie stereotypes held of groups that differ in status. In Fiske’s stereotype content model, stereotypes of groups that are higher in status tend to emphasize competence, whereas stereotypes of groups that are perceived to be competitors tend to be low in warmth [9]. As a result, stereotypes about many groups tend to be affectively mixed. People perceive those who are higher in social class to be higher in competence than warmth, whereas those who are lower in social class are perceived to be similar in terms of competence and warmth, or higher in warmth relative to competence [9]. However, direct comparison revealed that people with higher social class (and groups perceived to be higher in status) were perceived as more competent and though not as less warm (at least not consistently across studies) than those with lower social class. Although this work shed light on people’s stereotypes regarding social class and warmth and competence, lay theories regarding how social class affects other tendencies, such as social orientation (individualism vs. collectivism) or reasoning styles that do not easily fall along these two dimensions have not yet been assessed. In addition, this research does not answer the question of whether broader social class differences correspond to lay theories. Given the relative lack of cross-class interactions in contemporary American life, one would not be surprised if people’s lay beliefs regarding the consequences of social class do not fit neatly with the reality [10].

In the present set of studies I sought to systematically assess lay theories regarding the effects of social class across a variety of domains. Study 1 tested lay theories about the effect of class on psychological tendencies (individualism, contextualism, empathy, well-being), cognitive abilities (intelligence), and behavioral tendencies (honesty, conformity). Study 2 was a conceptual replication of Study 1, where participants rated members of different professions (High and Low Social Class Status) in terms of these 8 dimensions. Study 3 explored the accuracy of lay theories of social class effects on the Big Five dimensions of personality. Study 4 was a replication of Study 1 with the dimensions framed in opposite terms and included a comprehension check to ensure that participants understood correlations.

## Study 1

Study 1 explored the relationship between lay beliefs about how social class affects 8 broad dimensions (including psychological and behavioral tendencies, cognitive abilities, and health). Social class is positively correlated with 4 of the dimensions, and it is negatively correlated with the other dimensions. For each dimension participants were asked to indicate the correlation that they believed exists between social class and that dimension. If people’s beliefs about social class are accurate, then we would expect that they would predict correlations between class and the vast majority of these dimensions in the same direction as previous empirical studies (see Table 1 for sources and methods of computing empirical values [5,11-14],

**Table 1:** Additional demographic characteristics: Means (SDs), ranges.

	**Study 1**	**Study 2**	**Study 3**	**Study 4**	**Study 5**
**SSS**	4.91 (1.67)	5.32 (1.72)	4.95 (1.18)	5.00 (1.87)	4.77 (1.81)
range	1-9	2-10	1-10	1-10	1-9
**Education**	3.14 (1.01)	3.25 (1.06)	3.37 (1.11)	3.16 (1.15)	3.14 (1.15)
range	1-6	1-6	1-6	2-6	1-6
**Income^1^**	55.96 (43.26)	89.88 (90.76)	67.09 (74.58)	63.55 (48.33)	58.90 (48.25)
range	5-257	1-500	7-500	0-280	0-253

^1^ Income in thousands of US dollars.

To generate an empirical baseline for the relationship between social class and well-being, and between social class and health, US data from the World Values Survey wave 5 were used. A standardized composite of life satisfaction and happiness was created and was found to be significantly correlated with subjective social class (which was reverse scored so higher scores = higher class; N = 1179, *r* = .24, *p* < .001). Subjective health (reverse scored so higher scores = greater health) was also found to correlate with subjective social class (N = 1182, *r* = .23, *p* < .001).

### Methods

#### Ethics Statement

For all three studies informed consent was obtained. This research was approved by the local ethics committee of the Department of Psychology at Peking University.

#### Participants

One hundred participants (53f, 47m, Mage = 31.97, SD = 9.93, N = 97 US-born), completed the study online using the Qualtrics software program. Subjective social status (SSS) was assessed using the 10-point MacArthur subjective SES Ladder (SSS) [4,16], education was assessed on a 6 point scale (“1” = “did not complete HS,” “2” = “completed HS,” “3” = “Associate’s degree,” “4” = “BA or BS,” “5” = “MA or MS,” = “6,” “PhD, JD, or MD”), income was assessed in an open-ended fashion by having subjects indicate approximate annual income in thousands of dollars; a wide range of social class was represented in this sample (see Table 1). Participants were recruited through MTurk and paid $.50 to take part in a study that took only a few minutes. Only MTurk workers with ≥ 95% lifetime approval rate and who were also US residents were eligible to take part in this study or in the subsequent studies reported.

#### Procedure

Participants were asked to indicate the correlation between social class and 8 variables using slider bars (-1 to 1). The initial position of the bars was set to zero. Participants were given the following instructions:

When scientists study the relationship between different variables, say "*a*" and "*b*," these relationships can often be quantified in terms of a correlation coefficient known as "*r*."

***r*** can range from -1, a perfect negative correlation, meaning that for every increase of one unit in "*a*," there is a decrease by one unit of "*b*," to 1, a perfect positive correlation, meaning that for every increase in one unit of "*a*," there is an increase by one unit of "*b*." 0 means that the two variables are not correlated at all.

For the following questions we are interested in how strong you feel the correlation is between **social class** and several other variables. By social class, we mean a person’s overall status in society (a combination of wealth, education, power, and the prestige of their job). To indicate your answer, please slide the bar until it rests on the number you feel best represents the correlation between social class and that variable.

Definitions were provided for Individualism (“a sense of the self as unique and independent”), Contextualism (“a tendency to explain events as the result of situations rather than individuals and to see events as caused by multiple factors”), Well-Being (“a sense of happiness and satisfaction with life in general”), and Empathy (“a sense of concern for others and a sharing of their emotions”).

### Results

Across variables the average correlation predicted by participants was .27, SD = .49. Participants believed social class was positively correlated with all 8 variables. In 7 cases the mean correlation predicted was significantly different from zero, ts(99) ranging from 4.01–11.92, *p*s < .001. In the case of Honesty the mean was marginally different from zero, *t*(99) = 1.70, *p* = .09. Lay beliefs about social class were significantly different from actual correlations between social class and 7 of 8 variables (Table 2).

**Table 2:** Study 1: Lay beliefs about social class vs. actual findings.

	*r* with social class (mean lay belief)	*r* with social class (actual)	Difference between lay belief and actual
Individualism^1^	.22	.20	*t*(99) = .35, ns
Contextualism^1^	.20	-.23	*t*(99) = 10.00***
Well-Being^2^	.48	.24	*t*(99) = 5.79***
Empathy^3^	.21	-.28	*t*(99) = 10.08***
Intelligence^4^	.30	.55	*t*(99) = 4.83***
Health^5^	.49	.23	*t*(99) = 6.31***
Honesty^6^	.08	-.22	*t*(99) = 6.24***
Conformity^7^	.20	-.27	*t*(99) = 9.33***

^1^ Na et al., 2010, rs converted from ds for mean difference between high and low education groups on standardized composite scores for social orientation and cognitive style; ^2^ World Values Survey (WVS), wave 5 US data, r subjective social class and standardized composite score for happiness and life satisfaction; ^3^ Stellar et al., 2012, mean r of studies 1 (subjective social class), 2 (combined parental education and income), and 3 (combined parental education and income), converted from βs, studies 2 and 3 βs provided by author; ^4^ Nessier et al., 1996, correlation between years of education and IQ; ^5^ WVS wave 5 US data, r subjective health and subjective social class; ^6^ Piff et al., 2012, mean r across studies 3 (subjective social status), 4 (manipulated subjective social status), 5-7 (subjective social status), converted from *t*-values and βs; ^7^ Stephens et al., 2007, mean r across studies 1 (parental education), 2 (parental education), 4a (occupation), 4b (parental education), converted from χ^2^ s and *t*-values. *** *p* < .001.

The results did not appear to be driven by participants’ own social class as the only significant correlation between indicators of participants’ social class (SSS, education, income) was between income and believing social class to be correlated with Honesty (*r* = .18, *p* = .07) and this relationship was marginal.

## Study 2

Given that estimating correlations might be an unfamiliar exercise for lay people (despite fairly detailed instructions), a conceptual replication was performed in Study 2 with a more concrete task. Participants were asked to rate the average member of 8 occupations (4 high status, 4 low status) on each of the 8 dimensions used in Study 1.

### Methods

#### Participants

One hundred and fourteen participants (N = 114, 102 provided demographic information, 46f, 56m, Mage = 31.53, SD = 10.85, N = 95 US-born) completed the study online using the Qualtrics software program. As in Study 1 a wide range of social class was represented (additional demographic information presented in Table 1). Participants were recruited through MTurk and paid $.50 to take part in a study that took only a few minutes.

#### Procedure

Participants were asked to rate the extent to which the average member of 8 professions (4 high status, i.e. “lawyer,” “professor,” “doctor,” “banker,” 4 low status, i.e. “factory worker,” “firefighter,” “taxi driver,” “construction worker”) possessed the same 8 characteristics from Study 1 on 7-pt Likert scales (1 = very low, 7 = very high). The order in which professions appeared was randomized. Participants’ SSS was also measured.

### Results

**Figure 1:**
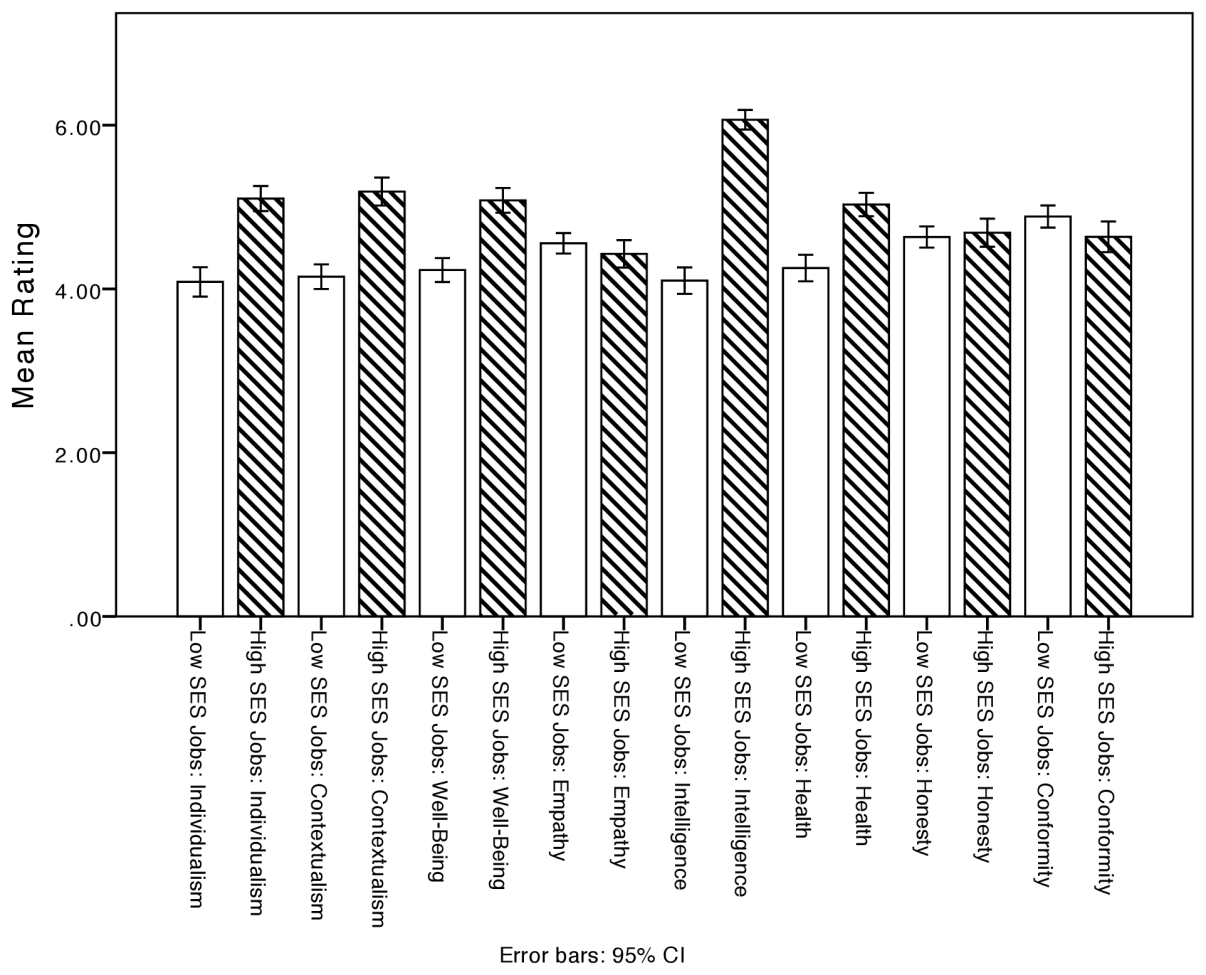
Study 2: Means and 95% Confidence Intervals for average ratings of high and low status professions.

Study 2 yielded highly similar results to Study 1. Repeated measures ANOVAs revealed that high status occupations on average were rated higher than low status occupations on 6 of 8 variables (see Figure 1), 5 of these differences were significant, Individualism: *F*(1,113) = 119.13, *p* < .001; Contextualism: *F*(1,113) = 79.28, *p* < .001; SWB: *F*(1,113) = 90.36, *p* < .001; IQ: *F*(1,113) = 345.13, *p* < .001; Health: *F*(1,113) = 68.68, *p* < .001. Only Conformity was rated higher for low status occupations, *F*(1,113) = 5.30, *p* = .02.

In order to test whether these effects were qualified by participants’ own social class, repeated measures multiple regression was performed using GLM. There were no significant interactions between participants’ social class and their beliefs about the tendencies of people with high vs. low status occupations, Multivariate *F*s < 1.55, ns.

## Study 3

Study 3 sought to test lay theories regarding the relationship of class and personality. Following the same procedure as in Study 1, participants were asked to indicate the correlation that they believed existed between social class and each of the Big 5 personality traits.

As no previously published large scale study assessed the simple correlations between social class and the Big Five (although a report exists comparing quintiles on social class and the Big Five [15]), data from the MIDUS survey (N = 2525) were used to provide an empirical baseline. Data from the most recent wave of the survey was used to compute these correlations. A standardized composite score of education, income, and occupational prestige was used as an indicator of social class. With the exception of Extraversion, social class was significantly correlated with each of the Big Five (see Table 2), *p*s < .001. Three of the traits were positively correlated with social class (two significantly so) and two were negatively correlated.

### Methods

#### Participants

One hundred and five participants (N = 105, 47f, 57m, 1 demographic data missing, Mage = 32.09, SD = 10.76, N = 98 US-born) completed the study online using the Qualtrics software program. As in Stuides 1 and 2, a wide range of social class was represented (additional demographic information presented in Table 1). Participants were recruited through MTurk and paid $.50 to take part in a study that took only a few minutes.

#### Procedure

Participants received the same instructions as in Study 1. They were asked to complete the same task with the Big Five personality dimensions. A definition was provided for each dimension adapted from Wikipedia, in order to make sure the definitions were accessible (Openness, “appreciation for art, emotion, adventure, unusual ideas, curiosity, and variety of experience;” Conscientiousness, “a tendency to show self-discipline, act dutifully, and aim for achievement; planned rather than spontaneous behavior;” Extraversion, “energy, positive emotions, and the tendency to seek stimulation in the company of others,” Agreeableness, “a tendency to be compassionate and cooperative rather than suspicious and antagonistic toward others;” Neuroticism, “a tendency to experience unpleasant emotions easily, such as anger, anxiety, depression, or vulnerability”). Participants’ SSS was also measured.

### Results

Across variables the average correlation predicted by participants was .15, SD = .48. Participants believed 4 of 5 traits were positively correlated with social class, 3 of which (Openness, Conscientiousness, and Extraversion) were significantly different from zero, ts(104) ranging from 3.62-8.34, *p*s < .001 (Table 3). Lay beliefs about social class were significantly different from actual correlations between social class and 4 of the 5 variables (Table 3).

**Table 3:** Study 3: Lay beliefs about social class vs. actual findings.

	*r* with social class (mean lay belief)	*r* with social class (actual)	Difference between lay belief and actual
Openness^1^	.34	.16	*t*(104) = 4.43***
Conscientiousness^1^	.27	.08	*t*(104) = 4.18***
Extraversion^1^	.15	-.02	*t*(104) = 4.09***
Agreeableness^1^	.03	-.12	*t*(104) = 3.75***
Neuroticism^1^	-.06	-.08	*t*(104) = .47, ns

^1^ MIDUS data, correlation between Big 5 personality traits and standardized composite social class score (education, income, occupational prestige). *** *p* < .001.

Participants’ own education was correlated with believing social class to be negatively correlated with Conscientiousness (*r* = -.20, *p* = .04) and Agreeableness (*r* = -.21, *p* = .04). There were no other significant correlations between indicators of participants’ own social class and their beliefs regarding the relationship between social class and the Big Five.

## Study 4

Given the fact that across studies participants believed that social class was positively correlated with the vast majority of dimensions assessed, and given the fact that in Study 1 people believed that social class was positively correlated with conceptually opposite (though arguably orthogonal) tendencies such as individualism and conformity, replication of Study 1 was performed with where the attributes were oppositely framed. Further, to ensure that the results were not due to a failure of participants to understand correlations (and as a safeguard against random responding), a comprehension check was also included.

### Methods

#### Participants

One hundred one participants took part in the study. Eight participants failed to answer all three comprehension check questions correctly (or failed to answer any) and were excluded from the final sample. The final sample consisted of 93 participants recruited through Amazon’s MTurk (N = 93, 24f, 69m, Mage = 33.35, SD = 11.76, 91 US-born), who were paid $.50 to complete an online study taking a few minutes.

#### Procedure

The same procedure was followed as in Study 1, however the opposite of each characteristic was used (“Individualism” → “Collectivism,” “Contextualism” → “Dispositionism,” “Well-Being” → “Distress,” “Empathy” → “Indifference,” “Intelligence” → “Stupidity,” “Health” → “Illness,” “Honesty” → “Dishonesty,” “Conformity” → “Uniqueness”). Definitions were provided for Collectivism (“a sense of the self as connected to and including close others and an emphasis on relationships”), Dispositionism (a tendency to explain events as the result of individuals rather than situations and to see events as caused by single factors), Distress (a sense of unhappiness and dissatisfaction with life in general), and Indifference (a lack of concern for others and a tendency not to share their emotions).

The main portion of the study was followed by a comprehension check in which subjects were asked three questions about correlations to ensure that they understood the meaning of positive, negative, and zero correlations (see Appendix S1).

### Results

Across variables the average correlation predicted between the 8 characteristics and social class was - .02 (SD = .21). In 5 cases the mean correlation predicted was significantly different from zero, ts(92) > 2.11, *p*s < .05 (see Table 4 for rs). The mean correlation predicted between social class and Uniqueness was marginally different from zero, *t*(92) = 1.81, *p* = .07, and mean correlations predicted between social class and Indifference and Dishonesty did not differ from zero, ts(92) < 1.27, ns. Lay beliefs about social class were significantly different from the actual correlations between social class and 6 of the 8 variables (Table 4).

**Table 4:** Study 4: Lay beliefs about social class vs. actual findings.

	*r* with social class (mean lay belief)	*r* with social class (actual)^1^	Difference between lay belief and actual
Collectivism	.20	-.20	*t*(92) = 8.09***
Dispositionism	.15	.23	*t*(92) = 1.54, ns
Distress	-.11	-.24	*t*(92) = 2.29*
Indifference	.00	.28	*t*(92) = 5.00***
Stupidity	-.21	-.55	*t*(92) = 7.35***
Illness	-.20	-.23	*t*(92) = .51, ns
Dishonesty	-.06	.22	*t*(92) = 5.68***
Uniqueness	.09	.27	*t*(92) = 3.52**

^1^ Correlations derived from same sources used in Study 1 with signs reversed. * p < .05, ** p < .01, *** p < .001.

Consistent with Study 1 where participants predicted positive correlations between social class and Well-Being, Intelligence, and Health, in Study 4 participants predicted negative correlations between social class and Distress, Stupidity, and Illness. Interestingly, despite the fact that participants in Study 1 predicted positive correlations between social class and Individualism, Contextualism, and Conformity, participants in Study 4 predicted positive correlations between social class and Collectivism, Dispositionism, and Uniqueness.

The results were largely unaffected by indicators of participants’ own social class, however SSS was marginally correlated with believing social class to be correlated with Uniqueness (*r* = .17, *p* = .09), income was correlated with believing social class to be correlated with Distress (*r* = .21, *p* < .05), and education was negatively correlated with believing social class to be correlated with Stupidity (*r* = -.23, *p* = .03) and Illness (*r* = -.25, *p* = .02).

## Study 5

Given the contradictory results of Study 1 and Study 4 on Individualism-Collectivism, Contextualism-Dispositionism, and Conformity-Uniqueness, a fifth study was conducted in which both versions of all 8 dimensions were presented, in order to assess whether the results reflect contradictory stereotypes regarding class or framing effects.

### Methods

#### Participants

Ninety-Nine participants took part in the study. Three participants failed to answer all three comprehension check questions correctly (or failed to answer any) and were excluded from the final sample. The final sample consisted of 96 participants recruited through Amazon’s MTurk (N = 96, 38f, 58m, Mage = 30.53, SD = 10.08, 85 US-born), who were paid $.50 to complete an online study taking a few minutes.

#### Procedure

The same procedure was followed as in Study 4, except that the 8 dimensions framed as they were in Study 1 were also included for a total of 16 items. The items were interspersed in the order presented in Appendix S2.

### Results

Across variables the average correlation predicted between the 16 characteristics and social class was .11 (SD = .22). The average correlation predicted between class and each of the 16 variables was significant for all variables (ts > 2.02, *p*s < .05) except Indifference (*t*(95) = .52, ns), Dishonesty (*t*(95) = 1.98, *p* = .05), and Contextualism (*t*(95) = 1.86, *p* < .07) which were marginally significant. Lay beliefs about social class were significantly different from the actual correlations between social class and 13 of the 16 variables (see Table 5). Each mean correlation replicated the results obtained previously in terms of direction (see Table 1, Table 4, and Table 5).

**Table 5:** Study 5: Lay beliefs about social class vs. actual findings.

	*r* with social class (mean lay belief)	*r* with social class (actual)^1^	Difference between lay belief and actual
Individualism	.23	.20	*t*(95) = .42, ns
Contextualism	.08	-.23	*t*(95) = 7.45***
Well-Being	.45	.24	*t*(95) = 4.67***
Empathy	.08	-.28	*t*(95) = 8.96***
Intelligence	.39	.55	*t*(95) = 3.85***
Health	.35	.23	*t*(95) = 2.82**
Honesty	.17	-.22	*t*(95) = 8.89***
Conformity	.20	-.27	*t*(95) = 10.82***
Collectivism	.32	-.20	*t*(95) = 12.20***
Dispositionism	.11	.23	*t*(95) = 2.87**
Distress	-.24	-.24	*t*(95) = .03, ns
Indifference	.02	.28	*t*(95) = 5.37***
Stupidity	-.25	-.55	*t*(95) = 6.19***
Illness	-.25	-.23	*t*(95) = .40, ns
Dishonesty	-.09	.22	*t*(95) = 6.85***
Uniqueness	.16	.27	*t*(95) = 2.82**

^1^ Correlations derived from same sources used in Study 1 and Study 4. * p < .05, ** p < .01, *** p < .001.

For the most part participants’ social class did not affect their lay beliefs regarding the consequences of social class. However, SSS was marginally correlated with believing social class to be negatively correlated with Empathy (*r* = -.18, *p* < .09), education was correlated with believing class to be negatively correlated with Collectivism (*r* = -.23, *p* < .03), positively correlated with Well-Being (*r* = .24, *p* = .02), and marginally positively correlated with Dispositionism (*r* = .19, *p* = .06). Participants’ income was not correlated with their lay beliefs.

At the individual-level lay beliefs regarding social class and Collectivsm and social class and Individualism were negatively correlated (*r* = -.22, *p* < .04), as were lay beliefs regarding Conformity and Uniqueness (*r* = -.22, *p* < .04) and Contextualism and Dispositionism (*r* = -.27, *p* = .007). However, none of these correlations were particularly strong given that the variables represented the same dimensions framed in opposite terms (i.e. “Individualism” and “Collectivism”).

### General Discussion

Five studies explored lay beliefs about the consequences of social class on a diverse range of psychological and behavioral tendencies and traits. These studies provide the first systematic description of lay beliefs about social class’ consequences for this broad variety of variables. They also provide the first systematic comparison of these beliefs with actual empirical findings. Across studies (Study 1, 3, 4) lay beliefs regarding social class were significantly different from the majority of empirical correlations between social class and those variables (17 out 21 variables). Even using a more generous criterion (whether mean lay beliefs shared the same sign as actual empirical correlations) lay beliefs corresponded only to 57% of the variables (12 out of 21).

The studies also revealed surprising results in terms of the consistency or coherence of lay beliefs about the consequences of social class. Although there was consistent evidence that participants linked higher social class to subjective well-being, intelligence, and health (Studies 1, 2, 4, and 5), lay beliefs about the links between social class and other variables appear more complex. For example, in Study 1 lay beliefs regarding social class and individualism and dispositionism were not significantly different from the actual correlations between social class and these variables. However, it is noteworthy that in Study 1 participants believed that social class was positively correlated with individualism, yet in Study 4 participants believed that social class and collectivism were also positively correlated. Similar findings were observed for contextualism and dispositionism, and conformity and uniqueness. In addition, participants predicted that all 6 of these variables were positively correlated with social class. These results seem puzzling, as it appears that people hold contradictory stereotypes about social class within the same domains. However, this pattern is not unique. As Allport noted, some stereotypes are “inherently contradictory” [17]. For example, there are prominent stereotypes of older adults as cognitively impaired and also as wise [18,19]. People also appear to hold contradictory beliefs about social class. For example, when asked how social class relates to conformity, people may associate wealth with manners and deference to tradition. Yet, asking how social class relates to uniqueness may activate schemas of people who are higher in status being more intent on self-expression and creativity compared to those who are lower in status. Similarly, contradictory results regarding social class and individualism vs. collectivism, may reflect the fact that people hold schemas of the rich as greedy and selfish (indicative of an individualist orientation) but also as more involved parents and as having more harmonious family relations (indicative of a collectivist orientation). People may hold contradictory stereotypes regarding how social class is linked to these domains, and how the domains are framed may cause different (contradictory) schemas to be accessible.

An alternative interpretation of the contradictory results of Studies 1 and 4 for individualism-collectivism, contextualism-dispositionism, and conformity-uniqueness is that they may reflect framing effects. However, the results of Study 5, which replicated the findings of both Studies 1 and 4 suggests that this is not likely to be the case.

It should also be noted that while the results of Study 5 confirmed that at the aggregate level there are contradictory lay beliefs regarding how social class is related to individualism vs. collectivism, uniqueness vs. conformity, and dispositionism vs. situationism, at the individual-level significant negative correlations were present. However, these correlations were much weaker than one might expect (| rs | < .28) for such diametrically opposed terms (i.e. “individualism” vs. “collectivism”), suggesting that even at the individual-level these beliefs are not particularly coherent.

Participants in Study 4 held lay beliefs about social class and indifference and dishonesty that mirrored those held by participants in Study 1 regarding social class and empathy and honesty, however in Study 4 the mean predicted correlations were not significantly different from zero, and as in Study 1 these predictions were contrary to the actual empirical relationship between social class and these variables. Study 5 yielded similar results. A recent Pew survey [20] found that 34% of people believe the rich are less likely than the average person to be honest (and only 12% believed they were more likely), and 55% indicated that they were more likely to be greedy (and only 7% believed they were less likely). Although at a glance these findings seem to stand in contrast to those of the current study, it is worth noting that 54% of respondents in the Pew survey either predicted no difference or had no opinion regarding whether the rich were more honest, and 36% responded in the same fashion to the question regarding the rich and greed. Taken together those results suggest fairly little perceived effect of social class on those variables, similar to the results of the present work.

In summary, although the proposition that stereotypes contain a grain of truth is often asserted, at least in the case of social class the stereotypes people hold in terms of a range of basic tendencies are not accurate. Why might people hold fairly accurate and consistent views of how social class relates to some variables but not others? If interactions and close relationships across social-class lines are fairly limited, then people might have less information about the reasoning styles (contextualism vs. dispositionism), personality traits (openness, conscientiousness, extraversion, agreeableness), and the relational (individualism vs. collectivism), social (conformity vs. uniqueness), and emotional (empathy vs. indifference) characteristics of those who do not share their social class position. In contrast, even limited interactions or observations are likely to allow for inferences about well-being (or distress), health (or illness) and also neuroticism (which is linked to anxiety and depressive symptoms), thus people may have more opportunities to develop relatively accurate schemas of how social class is linked to these particular variables.

Given that many of the dimensions assessed in this study can be seen as positive or desirable, one might suspect that the results reflect little more than a bias to view individuals with higher social class status as possessing more favorable traits. However, several facts argue against this interpretation. First, in Studies 2 and 4 participants did not predict significant effects of social class on honesty. Second, in Study 3 participants did not predict a significant correlation between social class and agreeableness. Third, in Study 3 although participants’ predictions regarding the correlation between class and neuroticism matched empirical data, the association that they predicted was not significantly different from zero.

The findings of the present study might also be interpreted through the prism of research on person perception. In a series of studies Chiu and colleagues [21] found that people who hold entity theories of personality (those who view traits as stable and fixed) are more likely to make trait inferences than those who hold incrementalist theories (those who view traits as dynamic and flexible across situations). The tendency to spontaneously infer traits also appears to be affected by culture, as European-Americans are more prone to engage in trait inference than East Asians [22]. Across studies participants predicted that a majority of traits and tendencies were associated in some way with class. This may reflect the fact that the samples were comprised of Americans (and predominantly European-Americans), it may also suggest that the majority of participants were entity theorists. Future research might explore the extent to which belief in entity theory in general, or specifically regarding class, may affect people’s lay theories of how class affects psychological and behavioral tendencies (and whether or not class is believed to be linked to these tendencies at all). It would also be interesting to know whether people are more likely to infer traits about high vs. low status individuals.

Interestingly, people’s own social class did not tend to affect their views about the relationship between class and the variables assessed in this study. This suggests that lay theories about the basic effects of social class may be fairly uniform in American society. These findings are also interesting in relation to Fiske’s stereotype content theory, as in the present studies lay theories linked high status to both greater competence (intelligence) and warmth (empathy). Although this pattern fits with Fiske’s data on how people view in-groups, given the socio-economic diversity of the current samples (both in terms of objective and subjective indicators of social class) and the general lack of an impact of participants’ own social class on their lay beliefs, it does not seem likely that the present findings reflect in-group bias. In fact, the few instances where social class had an effect on lay beliefs also do not neatly conform to this interpretation; while people with higher educational attainment thought there was a stronger negative correlation between social class and stupidity (Study 4), they also believed that social class was negatively correlated with both conscientiousness and agreeableness (Study 3).

It should be noted that in the present study participants were not asked specifically about the relationship between social class and either warmth or competence, so perhaps different results might have been observed if these dimensions had been measured directly. It would also be interesting to see if manipulating people’s sense that either those who were higher or lower in social class were competitors, which Fiske holds to be key in how out-groups are evaluated, might shift lay beliefs on the dimensions measured in this study in ways that are consistent with stereotype content theory.

Although the present study provides a description of lay beliefs regarding the effects of social class on a number of dimensions, social class is a multi-faceted construct. Thus it may be that people hold somewhat different stereotypes regarding the effects of say education and occupational prestige. This question is worth pursuing in the future and will provide a more nuanced understanding of social class-related stereotypes.

Finally, it should be noted that this research was conducted with American participants. Social class may have similar or different effects in other societies, and people’s lay theories (and the accuracy of such theories) might be different as well. These are empirical questions, and they should be explored in future research.

## Supporting Information

Appendix S1 Study 4: Comprehension Check. (DOCX)

Appendix S2 Study 5: Item Order. (DOCX)
