# Supplementary material for: What Are Lay Theories of Social Class?
Source: PLoS One. 2013 Jul 16;8(7):e70589. doi: 10.1371/journal.pone.0070589 (PMC3713045; doi:10.1371/journal.pone.0070589)
Supplement: Appendix S1 — (DOCX) [file pone.0070589.s001.docx]

Appendix S1.

Study 4: Comprehension Check

Which of the following correlations represents a negative relationship between two variables?

🞆 .5

🞆 0

🞆 -.6

Which of the following correlations represents a positive relationship between two variables?

🞆 0

🞆 .4

🞆 -.3

Which of the following correlations represents the lack of any relationship between two variables?

🞆 .9

🞆 0

🞆 -.9
