# Supplementary material for: What Are Lay Theories of Social Class?
Source: PLoS One. 2013 Jul 16;8(7):e70589. doi: 10.1371/journal.pone.0070589 (PMC3713045; doi:10.1371/journal.pone.0070589)
Supplement: Appendix S2 — (DOCX) [file pone.0070589.s002.docx]

Appendix S2.

Study 5 item order

1. Conformity
2. Collectivism
3. Dispositionism
4. Well-being
5. Intelligence
6. Distress
7. Empathy
8. Illness
9. Individualism
10. Indifference
11. Dishonesty
12. Contextualism
13. Uniqueness
14. Stupidity
15. Health
16. Honesty
